# Multiplex gene and phenotype network to characterize shared genetic pathways of epilepsy and autism

**DOI:** 10.1038/s41598-020-78654-y

**Published:** 2021-01-13

**Authors:** Jacqueline Peng, Yunyun Zhou, Kai Wang

**Affiliations:** 1 School of Engineering and Applied Science, University of Pennsylvania, Philadelphia, PA 19104 USA; 2 Raymond G. Perelman Center for Cellular and Molecular Therapeutics, Children’s Hospital of Philadelphia, Philadelphia, PA 19104 USA; 3 Department of Pathology and Laboratory Medicine, University of Pennsylvania, Philadelphia, PA 19104 USA

**Keywords:** Computational neuroscience, Data integration, Functional clustering, Computational biology and bioinformatics

## Abstract

It is well established that epilepsy and autism spectrum disorder (ASD) commonly co-occur; however, the underlying biological mechanisms of the co-occurence from their genetic susceptibility are not well understood. Our aim in this study is to characterize genetic modules of subgroups of epilepsy and autism genes that have similar phenotypic manifestations and biological functions. We first integrate a large number of expert-compiled and well-established epilepsy- and ASD-associated genes in a multiplex network, where one layer is connected through protein–protein interaction (PPI) and the other layer through gene-phenotype associations. We identify two modules in the multiplex network, which are significantly enriched in genes associated with both epilepsy and autism as well as genes highly expressed in brain tissues. We find that the first module, which represents the Gene Ontology category of ion transmembrane transport, is more epilepsy-focused, while the second module, representing synaptic signaling, is more ASD-focused. However, because of their enrichment in common genes and association with both epilepsy and ASD phenotypes, these modules point to genetic etiologies and biological processes shared between specific subtypes of epilepsy and ASD. Finally, we use our analysis to prioritize new candidate genes for epilepsy (i.e. *ANK2*, *CACNA1E*, *CACNA2D3*, *GRIA2, DLG4*) for further validation. The analytical approaches in our study can be applied to similar studies in the future to investigate the genetic connections between different human diseases.

## Introduction

Epilepsy and autism spectrum disorder (ASD) are two broad categories of brain disorders that are each characterized by substantial variability in the range of their clinical symptoms and strong but heterogeneous genetic association signals^1–10^. Epilepsy is a neurological disease characterized by recurrent seizures of different types. It is estimated that as much as 70% of epilepsies could have a strong genetic basis due to genetic defects^11^. ASD represents a complex range of neurodevelopmental conditions characterized by challenges in social interaction, nonverbal communication and repetitive behavior, each with varying degrees of impairment^12^. The heritability of ASD is estimated to be 56%-95% in various studies^13–15^, suggesting a strong genetic basis in ASD. Hereafter in the manuscript, we may use autism and ASD interchangeably for convenience.

There is a surprisingly high co-occurrence of these two disorders, for example, about 2–3% of children are estimated to have epilepsy, yet that percentage rises to around 30% in autism cases^16,17^. One current hypothesis is that the co-occurrence of epilepsy and autism stems from the disruption of shared neurodevelopmental pathways implicated by the relatively high number of genes associated with both disorders^18,19^. Certain biological pathways are involved in both disease processes, such as transcription regulation, cellular growth, and synaptic regulation^20^. The excitation/inhibition balance hypothesis suggests that neurodevelopmental defects, primarily of GABAergic and glutamatergic systems, lead to an imbalance in excitatory and inhibitory neural circuits that lead to the pathogenesis of both disorders^21^. However, the exact mechanisms involved in these two disorders still need to be further elucidated^24^.

Several studies using network-based approaches have been taken to identify shared pathways and candidate genes for epilepsy or autism^22–27^. These studies generally use protein–protein interaction (PPI), co-expression, shared biological processes/pathways, and other networks to represent relationships between autism- or epilepsy-associated genes from sequencing data or curated databases. However, few of them have studied epilepsy and autism within the context of each other. In one such study, a random walk-based clustering approach was used to dissect modules of highly interacting genes representing epilepsy phenotypes from genes more generally involved in neurodevelopmental disorders^22^. Furthermore, epilepsy genes were predicted based on these modules. While the assumption is that genes in highly connected biological modules are likely to manifest similar phenotypes^28^, as far as we know, none or few epilepsy/autism network studies directly use phenotype networks based on gene-phenotype associations. Phenotype networks can potentially reveal additional information about the relationships among entities in the network, such as distinguishing genetic modules of primarily epilepsy-related phenotypes from those of autism-related phenotypes^29^.

**Figure 1:**
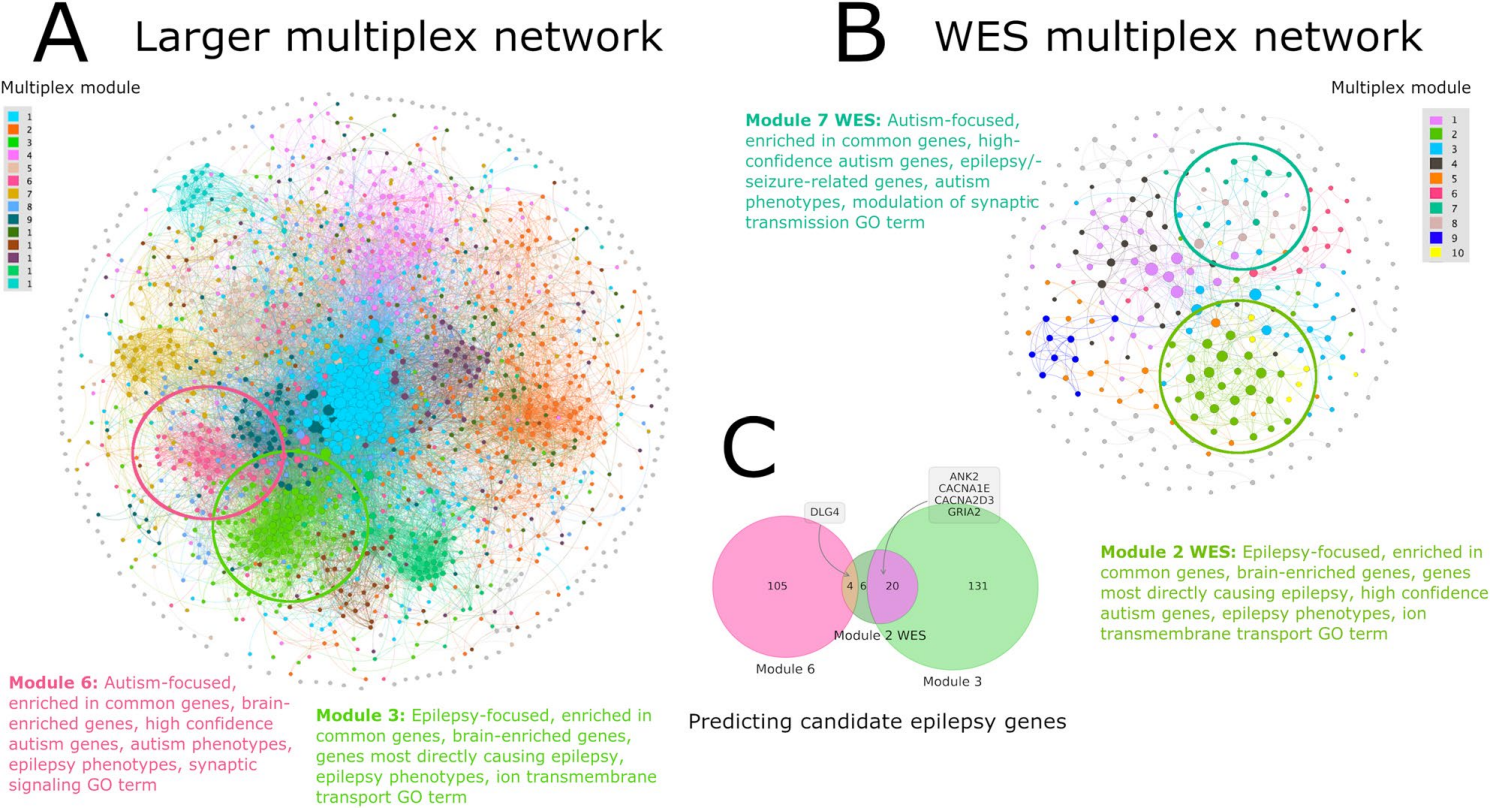
Summary of study findings. (**A**) A network of all 1707 epilepsy- and autism-associated genes from Wang et al. (2017)^10^ and SFARI^60^ and (**B**) A network of 294 epilepsy and autism genes from WES studies^38,61^. The edges represent the union of edges from the PPI network layer and phenotype network layer of the multiplex network. The color of a node represents the module it belongs to in the multiplex network and the size of the node is relative to its degree in the network. Key modules identified in the study are annotated. The network plots were generated using Gephi version 0.9.2, a graph visualization software. (**C**) The five autism-specific genes (i.e. not listed in Wang et al. (2017)^10^) in module 3 and 6 of the larger multiplex network that overlap with epilepsy-focused module 2 of the WES network are predicted candidate epilepsy genes.

This present project is motivated not only by the high co-morbidity rate of these two diseases, but also by the frequent observation that when a new candidate gene is published for one disease, it is often already a well-known gene for another disease. Our central hypothesis is that the genetic overlap is due to high heterogeneity of both diseases, so there will be autism-specific, epilepsy-specific and shared genetic modules, and studying each type of module allows us to identify novel candidate genes for each disease and characterize their phenotypic consequences. Therefore, in this present study, our aim is to characterize genetic modules and prioritize candidate genes for both disorders, taking advantage of the large number of expert-compiled and well-established disease-associated genes for each of the diseases. First, we constructed a phenotype network of epilepsy- and autism-associated genes using gene-phenotype associations from the comprehensive database of Phen2Gene, a phenotype-drive gene prioritization tool^30^. We then integrated the protein–protein interaction (PPI) relationships of these genes using a multilayer network. Then, we used a community detection algorithm to identify modules of highly interacting genes with similar phenotypic manifestations in the multiplex network. While previous network-based studies have focused primarily on studying either epilepsy or autism genes, the novelty in this study comes from analyzing both epilepsy- and autism-associated genes together in a multiplex network, formally taking both PPI interactions and gene-phenotype associations into consideration. As the result of our study, we prioritized two modules enriched in common genes, genes associated with both epilepsy and autism, representing the biological processes of ion transmembrane transport and synaptic signaling, respectively, that may contribute to the shared genetic etiology between epilepsy and autism. One of the two modules is an epilepsy-focused module enriched in genes directly causing epilepsy and epilepsy phenotypes and the other is an autism-focused module enriched in highest confidence autism genes and autism phenotypes. Additionally, we identified corresponding modules in a similar multiplex network constructed with solely epilepsy and autism genes from whole-exome sequencing (WES) studies. Finally, we prioritized candidate epilepsy genes based on the overlap of these prioritized modules. These findings are summarized in Fig. 1. Understanding the genetic connection of epilepsy and autism can aid in the discovery and prioritization of candidate genes for either disorder and the understanding of their shared molecular pathophysiology.

## Results

### Constructing a multiplex network from PPI and phenotype relationships between autism- and epilepsy-associated genes

From recently published review literature and from the SFARI (Simons Foundation Autism Research Initiative) database, we compiled 999 established epilepsy-associated genes and 913 known autism-associated genes to be used as nodes in the PPI, phenotype, and multilayer networks. Among them, 205 genes are shared by both groups (Fig. 3A), which we will subsequently refer to as “common genes”, so in total there were 1707 unique genes. We note that each of the two gene lists can be sub-divided into several subgroups based on varying levels of association or confidence (Table 1). For example, epilepsy genes that “only cause epilepsies or syndromes with epilepsy as the core symptom” are classified in subgroup 1, yet genes associated with “gross brain developmental malformations and epilepsies” are classified in subgroup 2. Similarly, SFARI genes are classified into distinct groups based on a scoring system that takes into account different sources of information reflecting the strength of the evidence linking it to the development of autism. Our subsequent analysis considers all epilepsy and autism subgroups.

**Table 1:** Summary of epilepsy- and autism-associated genes used in the current study. Gene clusters from the original epilepsy and autism gene lists were separated into individual genes and gene symbols were standardized; the counts shown were taken after this step.

Epilepsy subgroup	Number of genes	Classification by Wang et al. (2017)^10^
Epilepsy 1	84	“Epilepsy genes, i.e., genes that only cause epilepsies or syndromes with epilepsy as the core symptom”
Epilepsy2	73	“Neurodevelopment-associated genes, i.e., genes associated with gross brain developmental malformations and epilepsies”
Epilepsy 3	529	“Epilepsy-related genes, i.e., genes associated with gross physical, or other systemic abnormalities and accompanied by epilepsy or seizures”
Epilepsy 4	314	“Potential epilepsy-associated genes, i.e., genes that require further verification”

Autism subgroup	Number of genes	Classification by SFARI (accessed February 21, 2020)
Autism 1	144	Genes in this category are all found on the SPARK gene list. Each of these genes has been clearly implicated in ASD—typically by the presence of at least three de novo likely-gene-disrupting mutations being reported in the literature—and such mutations identified in the sequencing of the SPARK cohort are typically returned to the participants. Some of these gene meet the most rigorous threshold of genome-wide significance; all at least meet a threshold false discovery rate of < 0.1
Autism 2	219	Genes with two reported de novo likely-gene-disrupting mutations A gene uniquely implicated by a genome-wide association study, either reaching genome-wide significance or, if not, consistently replicated and accompanied by evidence that the risk variant has a functional effect
Autism 3	472	Genes with a single reported de novo likely-gene-disrupting mutation Evidence from a significant but unreplicated association study, or a series of rare inherited mutations for which there is not a rigorous statistical comparison with controls
Autism S	119	The syndromic category includes mutations that are associated with a substantial degree of increased risk and consistently linked to additional characteristics not required for an ASD diagnosis. If there is independent evidence implicating a gene in idiopathic ASD, it will be listed as “#S” (e.g., 2S, 3S). If there is no such independent evidence, the gene will be listed simply as “S”

**Figure 2:**
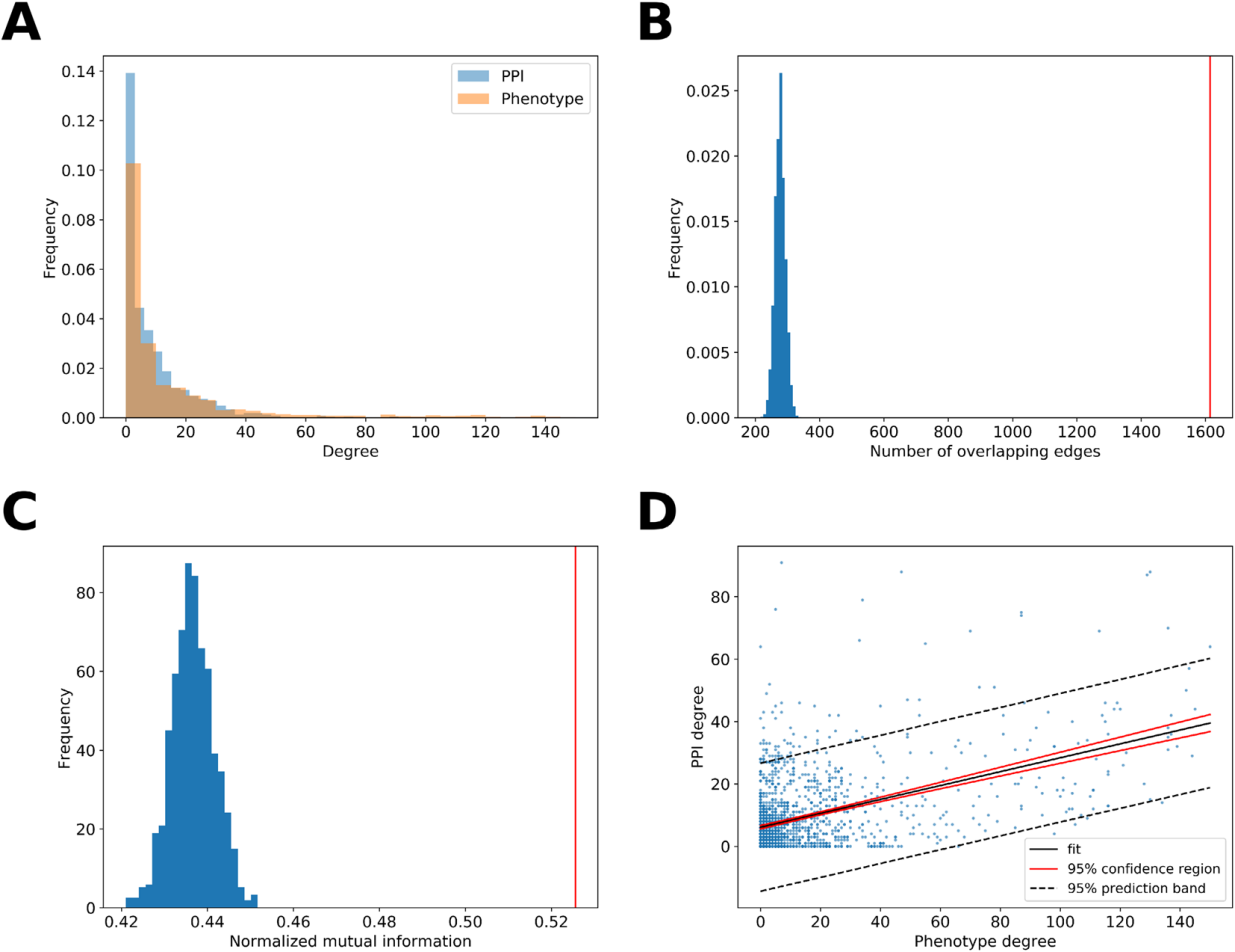
Similarity of the PPI and phenotype network layers in the epilepsy-autism multiplex network. (**A**) The degree distribution within each layer of the multiplex network is plotted. (**B**) There is a significant number of edges overlapping between the PPI network layer and phenotype network layer (p < 0.0001). The distribution represents the number of overlapping edges from 10,000 trials where the PPI network and phenotype network were randomly generated maintaining their original degree distribution. The red line represents the actual number of overlapping edges between the two networks. (**C**) There is a significant overlap in the modules in the PPI network layer and the modules in the phenotype network layer (p < 0.001). The distribution represents the normalized mutual information from 1000 trials where the PPI network and phenotype network were randomly generated maintaining their original degree distribution. The red line represents the actual normalized mutual information. (**D**) There is a correlation between the degree of a node in the PPI network layer and phenotype network layer. Each point represents one of the 1707 genes/nodes in the multiplex network.

**Figure 3:**
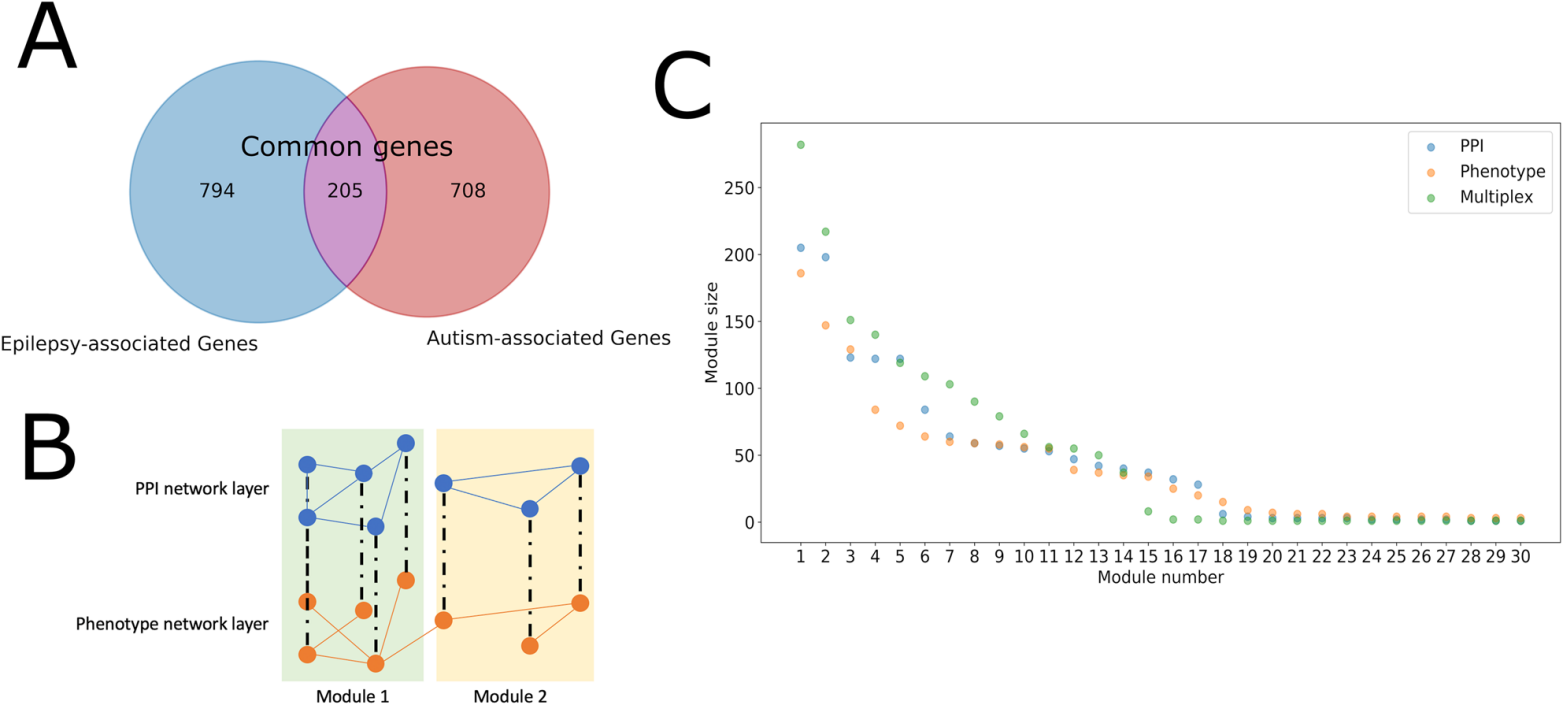
Summary of multiplex network construction and network modules. (**A**) In the epilepsy-autism gene network there are a total of 1707 genes represented, including 999 epilepsy-associated genes and 913 autism-associated genes (205 genes are shared). (**B**) Using the 1707 genes a multiplex gene network was created. One layer of the multiplex network represents protein–protein interactions (PPIs) between the genes retrieved from the STRING database. The other layer was created using gene-phenotype relations retrieved from the Phen2Gene knowledge base. A multiplex version of the well-known Louvain algorithm was applied on the multiplex network to generate modules taking both layers of the network into consideration. The regular Louvain algorithm was also applied to each layer separately to generate modules using only one layer. (**C**) The figure plots the size of the 30 largest modules generated from the PPI network layer, the phenotype network layer, and the multiplex network.

The 1707 epilepsy- and autism-associated genes and all 7903 edges between them from the STRING PPI network constituted one of two layers of the epilepsy-autism multiplex network (Fig. 3B). The same 1707 genes and all 12,070 edges between them from the gene-phenotype network constituted the other layer in the multiplex network. When analyzing both layers as individual networks, we found that their degree distributions were similar (Fig. 2A) and there was a significant (p < 0.0001) overlap in their edges compared to random networks with the same degree distribution (Fig. 2B). Moreover, after running the Louvain clustering algorithm on both networks, the normalized mutual information, which represents how similar the two partitions are, was significantly (p < 0.001) greater than random networks with the same degree distribution (Fig. 2C). There is also a positive correlation between a gene’s degree in the PPI network and its degree in the phenotype network (Fig. 2D). These results support that information from the gene-PPI network is consistent with that of the gene-phenotype network. The multiplex network was created by stacking the two layers such that the same genes in the two layers aligned. From the 1707 epilepsy- and autism-associated genes, there were 1556 genes that had a degree of at least one in either the gene-PPI or gene-phenotype layer.

We used the Louvain algorithm to partition the multiplex network (Fig. 1B). While there were 1707 genes in the network, only 17 modules contain at least two genes (Fig. 3C). We chose to focus on the 14 largest modules for the remainder of the analysis because of the large drop in number genes from module 14 (37 genes) to module 15 (8 genes). We also used the Louvain algorithm on the individual PPI and phenotype network layers. While the module sizes were similar among the two layers, the module sizes for multiplex partition were slightly larger, owing to the greater number of gene–gene relationships revealed when both layers were taken into account (Fig. 3C). In summary, the epilepsy and autism genes converge on a limited number of modules and we will show the biological significance of those modules in the next section.

### Gene set enrichment analysis of modules within the multiplex network

**Figure 4:**
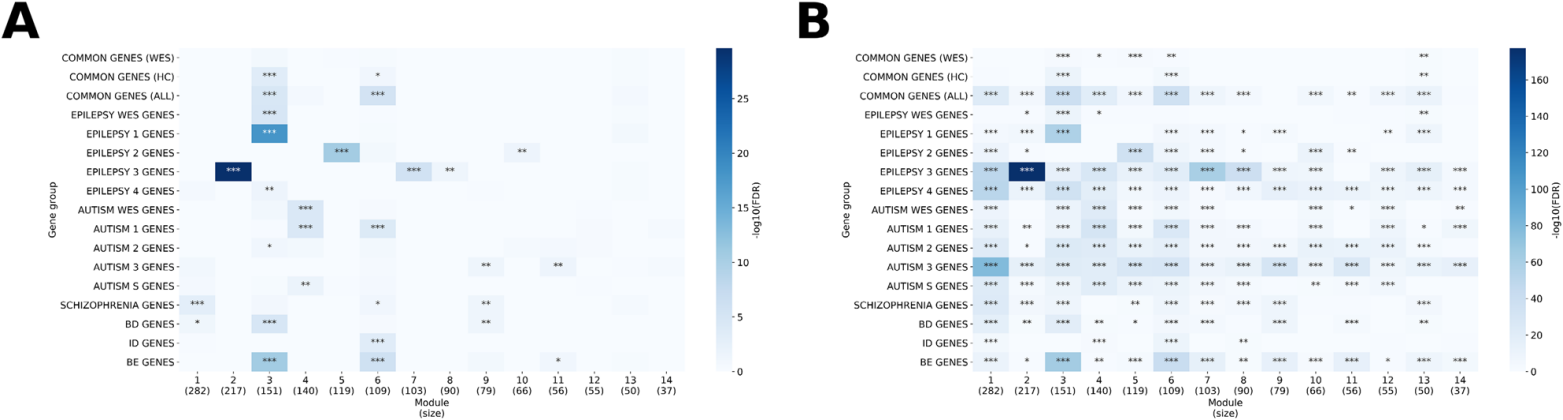
Gene enrichment analysis on modules in the epilepsy-autism multiplex network. The enrichment analysis of different gene groups over the 14 largest modules in the epilepsy-autism multiplex network. The hypergeometric test was used to determine the p-value and the false discovery rate (FDR) is reported since multiple gene groups were tested. (**A**) The background of the hypergeometric test is the 1707 genes in the network. (**B**) The background of the hypergeometric test is 19,556 genes (the number of genes in the STRING database). COMMON GENES (WES) = genes in both the epilepsy and autism WES gene lists, COMMON GENES (HC) = genes that are both in the epilepsy 1 subgroup and autism 1 subgroup (high confidence), COMMON GENES (ALL) = all genes in both  epilepsy subgroup and autism subgroup, BD = bipolar disorder, ID = intellectual disability, BE GENES = genes that have significantly higher expression in brain tissue vs control tissue. For both A) and B), “***” denotes FDR < 0.01, “**” denotes FDR < 0.05, and “*” denotes FDR < 0.1, assuming a hypergeometric distribution. The heatmap was generated using seaborn version 0.10.0 (https://seaborn.pydata.org/).

We focused on the 205 common genes that are associated with both epilepsy and autism and found that several modules tend to have significant enrichment of these genes (Fig. 4). Among the 14 largest modules in the multiplex network, modules 3, consisting of 151 genes, and module 6, consisting of 109 genes, are significantly enriched in common genes (FDR = 1.89e−05 and FDR = 2.99e−06). Furthermore, module 3 is enriched in high confidence (HC) common genes, which is the intersection of subgroup 1 of epilepsy and subgroup 1 of autism genes (FDR = 3.92e−04). The most significantly enriched biological process GO (Gene Ontology) term for these two modules are ion transmembrane transport (GO:0034220, FDR = 1.09e−82, 99/151 genes in the module) and synaptic signaling (GO:0099536, FDR = 4.83e−43, 52/109 genes in the module), respectively, for modules 3 and 6. The enrichment of common genes in these two modules indicate that they likely represent biological processes relevant to both epilepsy and autism.

In addition to GO-based functional enrichment analysis, we also explored whether there are specific patterns of tissue-specific gene expression in the modules that are enriched for pleotropic genes affecting both epilepsy and autism, specifically module 3 and 6. For this analysis, we utilized the RNA-Seq data of Genotype-Tissue Expression (GTEx) consortium to identify genes that tend to have higher expression levels in brain tissue than other tissues, which we will refer to as brain-enriched genes (BEGs). Among the 14 largest modules, only modules 3 (FDR = 1.74e−11) and module 6 (FDR = 9.59e−07) have a significant enrichment of BEGs (Fig. 4A) relative to other modules. We acknowledge that since both epilepsy and autism are brain diseases, it is expected that disease-relevant genes are likely brain-expressed genes a priori. As expected, we confirmed that nearly all BEGs in these two modules are upregulated genes. In summary, the relatively large number of BEGs and their highly significant enrichment further support the functional relevance of these two modules in the shared etiology between epilepsy and autism, and possibly other developmental disorders. In fact, module 3 was enriched in bipolar disorder (BD) genes (FDR = 1.69e−05), and module 6 was enriched in intellectual disability (ID) genes (FDR = 3.95e−04).

Next, we explored the relationship between subtypes of epilepsy and autism by considering the different subgroups of genes that are associated with epilepsy (subgroup 1, 2, 3, 4) and autism (subgroup 1, 2, 3 and S). Additional details on the classification of these subgroups are given in Table 1. The distribution of these subgroups within the 14 largest multiplex modules are shown in Fig. S2. We found that certain modules are enriched in specific subgroups of epilepsy genes (Fig. 4). Module 3, which represents the GO biological process of ion transmembrane transport as discussed previously, is enriched in Epilepsy 1 genes (genes directly causing epilepsy) compared to other modules (FDR = 7.14e−19). Module 3 is also the only module enriched in whole-exome sequencing (WES) epilepsy genes (FDR = 1.52e−05) and Epilepsy 4 genes (FDR = 0.0270) (predicted epilepsy genes) relative to other modules. Modules 5 and 10 are enriched in Epilepsy 2 genes (neurodevelopment-associated genes) compared to other modules (FDR = 2.58e−11 and FDR = 0.0255, respectively). The most significant biological process GO term for these two modules are cell cycle process (GO:0022402, FDR = 7.07e−29, 56/119 genes in the module) and RNA metabolic process (GO:0016070, FDR = 2.04e−12, 49/66 genes in the module), respectively. Modules 2, 7, and 8 are enriched in Epilepsy 3 genes (genes associated with other abnormalities and accompanied by epilepsy or seizures) compared to other modules (FDR = 2.37e−30, FDR = 2.00e−05, FDR = 0.0343, respectively). The most significant GO biological process for these three modules are organic acid metabolic process (GO:0006082, FDR = 2.85e−62, 96/217 genes in the module), carbohydrate derivative biosynthetic process (GO:1901137, FDR = 6.09e−34, 48/103 genes in the module), and vesicle-mediated transport (GO:0016192, FDR = 1.06e–05, 28/90 genes in the module) respectively. Therefore, module 3 and its associated GO biological functions likely have more of a direct relationship to epilepsy pathogenesis, while modules 2, 5, 7, 8, and 10, although epilepsy-related, have a more indirect relationship to epilepsy through neurodevelopment or other abnormalities. Table S2 in the supplementary Excel file contains additional information on the GO enrichments of each module as well as the subgroups of epilepsy and autism genes in the module.

Similarly, specific modules are enriched in specific subgroups of autism genes (Fig. 4). Unlike epilepsy, we note that the SFARI autism genes are classified based on confidence of association, given that autism is a complex neurodevelopmental disorder. Modules 4 and 6 are enriched in Autism 1 genes (autism genes with the highest confidence) compared to other modules (FDR = 4.30e−05 and FDR = 9.15e−05, respectively). The most significant GO biological process for these two modules, respectively, are chromatin organization (GO: 0006325, FDR = 7.45e−50, 68/140 genes in the module) and synaptic signaling, as discussed previously. Module 4 is also enriched in WES autism genes (FDR = 4.30e−05) and Autism S genes (syndromic autism genes) (FDR = 0.0152) relative to other modules. No module was relatively enriched in Autism 2 genes (strong autism candidates) with FDR < 0.05 although module 3 had the lowest FDR (FDR = 0.0821). Modules 9 and 11 were enriched in Autism 3 genes (genes with suggestive evidence) compared to other modules (FDR = 0.0467 and FDR = 0.0299, respectively). Therefore, modules 4 and 6 probably represent the set of most confident autism-associated genes and their associated GO biological functions give insight into autism pathogenesis.

### Phenotype enrichment analysis of modules within the multiplex network

**Figure 5:**
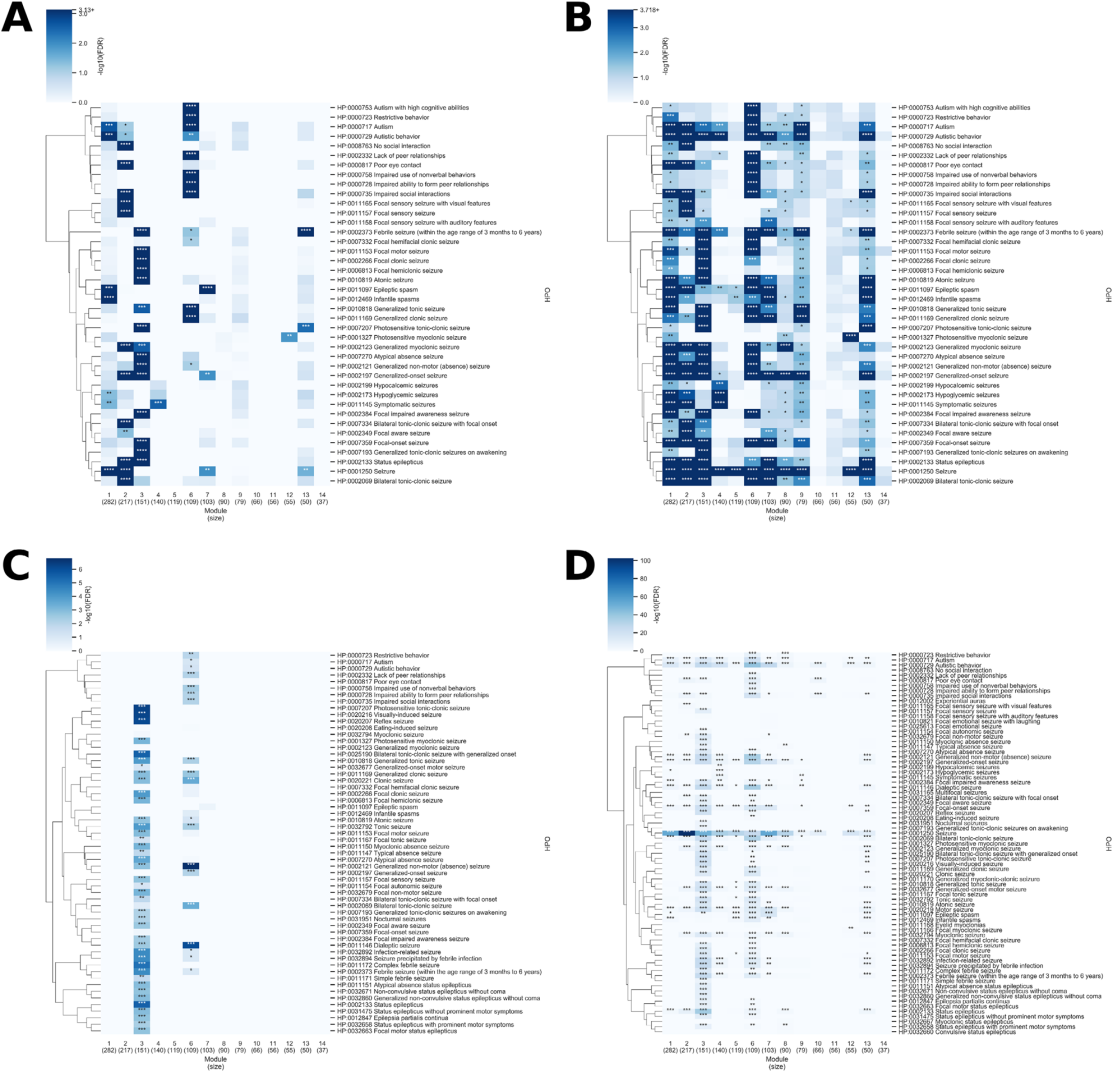
Enrichment analysis of epilepsy- and autism-related HPO terms for modules in the multiplex network. The enrichment of different epilepsy and autism phenotypes over the 14 largest modules in the epilepsy-autism multiplex network is shown. The first cluster of HPO terms represent autism phenotypes and the rest represent epilepsy phenotypes. Only HPO IDs with gene-HPO relationships in the Phen2Gene knowledgebase are shown. The p-value was determined by computing the mean gene-phenotype association score for each HPO ID over the genes in the module and comparing it to the mean of 10,000 trials using *n* genes, where *n* is the size of the module, randomly chosen from (**A**) the 1707 genes in the multiplex network or (**B**) all genes in the Phen2Gene knowledge base. (**C**) and (**D**) correspond to (**A**) and (**B**), respectively, except that the phenotype enrichment was calculated using annotated gene-HPO relationships from hpo.jax.org, The hypergeometric test was used to determine the p-value and the false discovery rate (FDR) is reported. For all plots, the false discovery rate (FDR) is reported since multiple HPO IDs were tested. “****” denotes FDR < 0.0001, “***” denotes FDR < 0.01, “**” denotes FDR < 0.05, and “*” denotes FDR < 0.1. The clustermap was generated using seaborn version 0.10.0 (https://seaborn.pydata.org/). The linkage on the rows was generated based on the distance between HPO IDs in the HPO tree.

Along with characterizing each module by enrichment in gene sets, we can also characterize them by enrichment in human phenotype ontology (HPO) associations^31^. Figure 3 displays the various HPO enrichments among the 14 largest multiplex modules. Module 3 is most significantly enriched in epilepsy phenotypes (HPO IDs in the HPO subtree with a root of HP:0001250–seizure) compared to other modules in the network (Fig. 5A,C). This is consistent with the module’s enrichment in Epilepsy 1 genes and WES epilepsy genes. Module 6 is mostly significantly enriched in autism phenotypes (HPO IDs in the HPO subtree with a root of HPO: HP:0000729—autistic behavior) compared to other modules in the network (Fig. 5A,D). This is consistent with the module’s enrichment in Autism 1 genes. While module 4 was also enriched in Autism 1 genes, as well as WES autism genes, the module was not enriched in any autism phenotypes relative to other modules in the network. This could be because the module is enriched in syndromic autism genes that are strongly associated with other phenotypes besides ASD, leading to a relatively lower gene-ASD phenotype association score. Interestingly, module 2 has enrichment in autism phenotypes despite only being enriched in Epilepsy 3 genes. Module 6, as well as being enriched in autism phenotypes, was also enriched in epilepsy phenotypes relative to other modules, which could be explained by the enrichment in common genes in the module. While module 3 was also enriched in common genes and enriched in epilepsy phenotypes, it was not enriched in autism phenotypes relative to other modules. However, when looking at enrichment relative to all genes, including those outside the network, most of the 14 largest modules have enrichment in both epilepsy and autism phenotypes (Fig. 5B,D). Thus, while module 6 is likely most representative of autism relative to the other modules because of its enrichment autism genes and phenotypes, it may also be relevant to epilepsy because of its enrichment in common genes, BEGs, and some epilepsy phenotypes. Similarly, while module 3 is most representative of epilepsy relative to the other modules because of its enrichment in epilepsy genes and phenotypes, its enrichment in common genes and BEGs suggest that it shares a genetic basis with autism. Table S2 in supplementary Excel file contains additional information on the HPO enrichments of each module.

### Analysis on WES epilepsy-autism multiplex network

**Figure 6:**
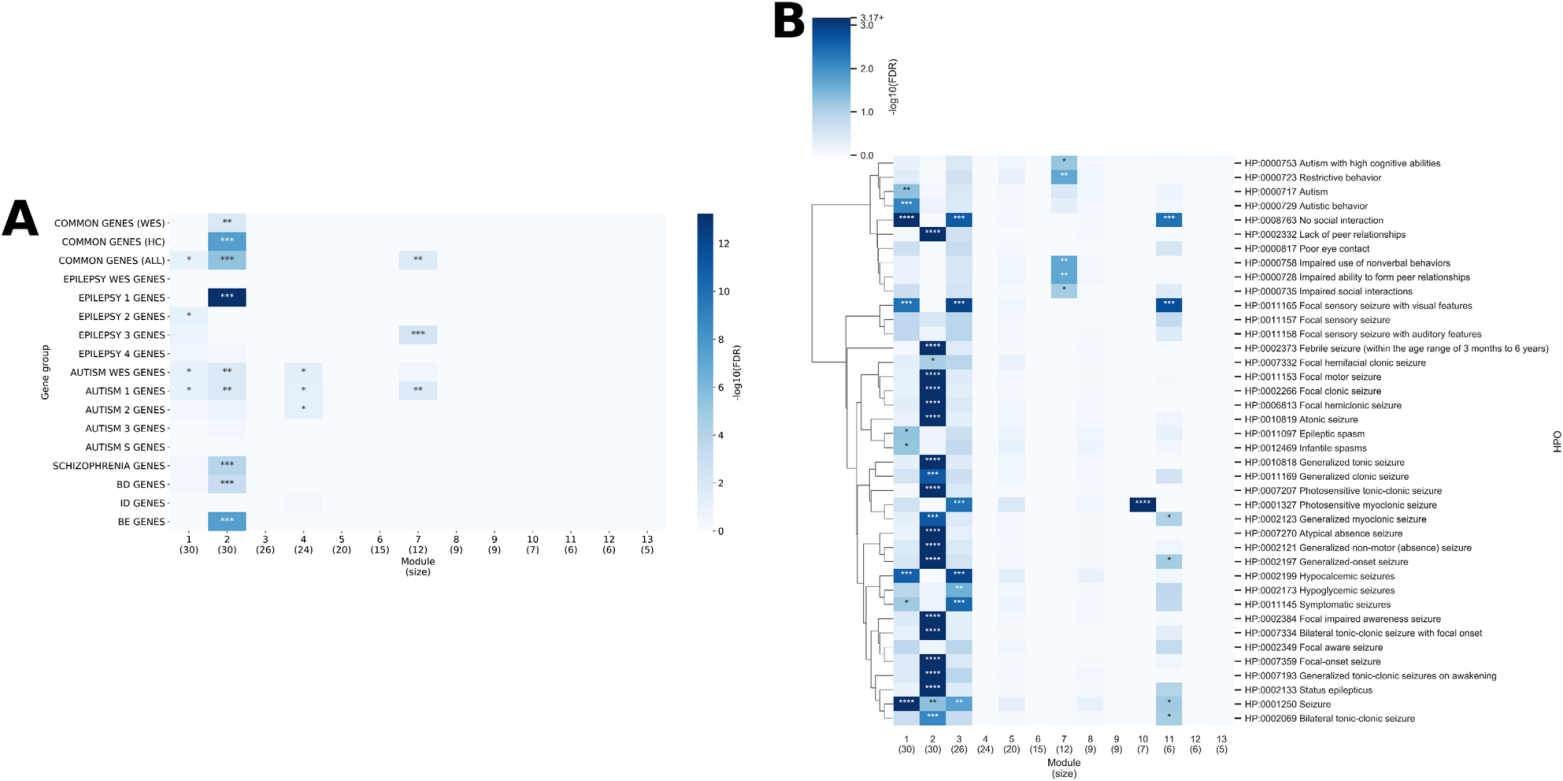
Enrichment analysis on modules in the multiplex network generated with WES epilepsy and autism genes. The enrichment analysis of (**A**) different gene groups and (**B**) epilepsy and autism phenotypes over the 13 largest modules (those that have at least 5 genes) in the multiplex network generated using only WES epilepsy and autism genes. (**A**) The hypergeometric test was used to determine the p-value for enrichment in each gene group. The false discovery rate (FDR) is reported since multiple gene groups were tested. The background of the hypergeometric test is the 294 genes in the network. COMMON GENES (WES) = genes in both the epilepsy and autism WES gene lists, COMMON GENES (HC) = genes that are both in the epilepsy 1 subgroup and autism 1 subgroup (high confidence), COMMON GENES (ALL) = all genes in an epilepsy subgroup and autism subgroup, BD = bipolar disorder, ID = intellectual disability, BE GENES = genes that have a significantly higher expression in brain tissue vs control tissue. (**B**) The first cluster of HPO terms represent autism phenotypes and the rest represent epilepsy phenotypes. Only HPO IDs with gene-HPO relationships in the Phen2Gene knowledgebase are shown. The p-value was determined by computing the mean gene-phenotype association score for each HPO ID over the genes in the module and comparing it to the mean of 10,000 trials using *n* genes, where *n* is the size of the module, randomly chosen from the 294 genes in the WES multiplex network. The FDR is reported since multiple HPO IDs were tested. For both (**A**) and (**B**) “***” denotes FDR < 0.01, “**” denotes FDR < 0.05, and “*” denotes FDR < 0.1 and for (**B**) “****” denotes FDR < 0.0001. The heatmap and cluster map were generated using seaborn version 0.10.0 (https://seaborn.pydata.org/). The linkage on the rows of the cluster map was generated based on the distance between HPO IDs in the HPO tree.

**Figure 7:**
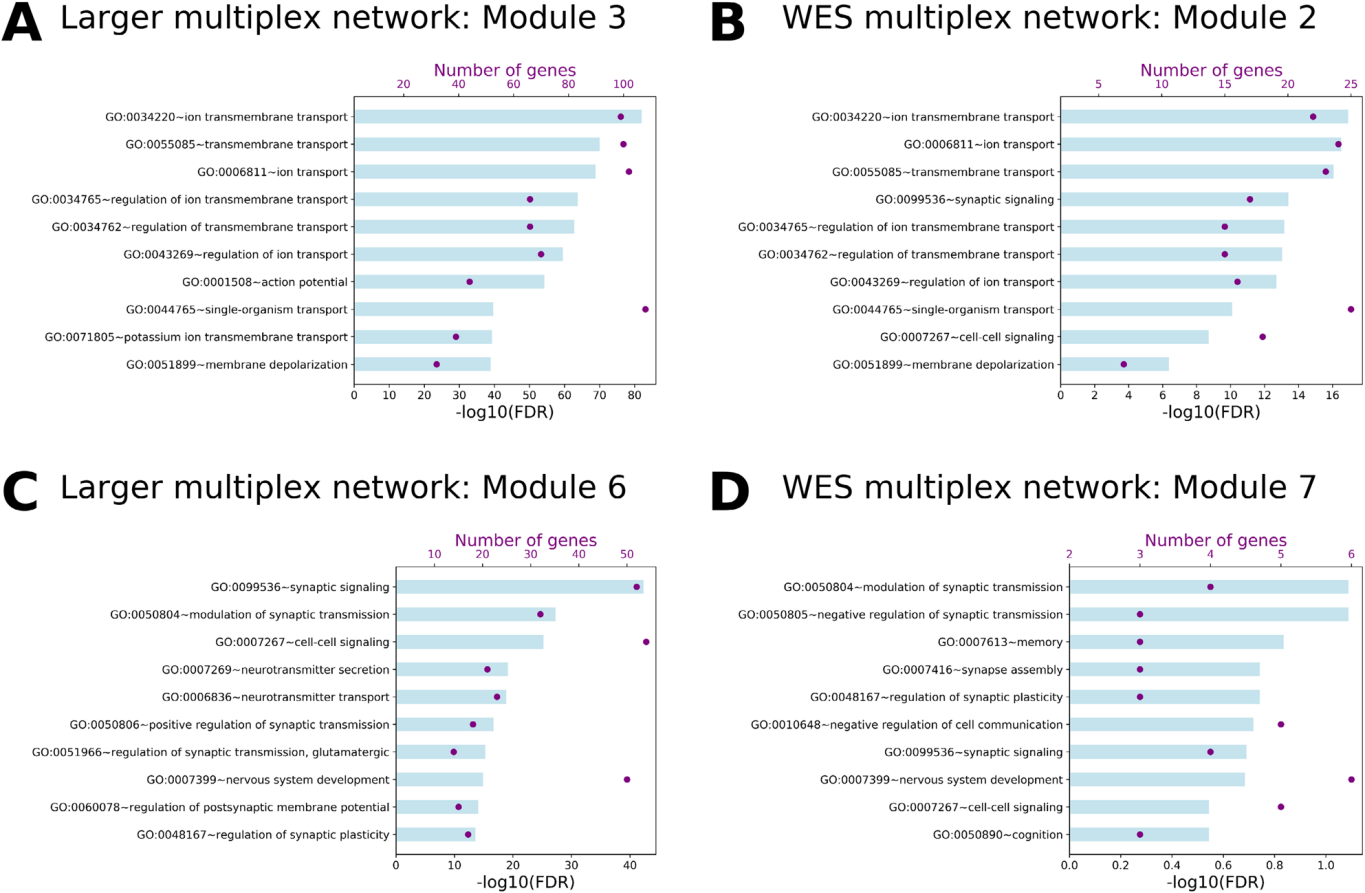
Comparison of prioritized modules in the WES multiplex network and larger epilepsy-autism multiplex network. The top 10 most significant biological process Gene Ontology terms by FDR are shown for (**A**) Module 3 and (**B**) Module 6 of the larger multiplex network and their corresponding modules (**C**) Module 2 and (**D**) Module 7 of the WES multiplex network.

All analyses were repeated with a multiplex network generated purely from epilepsy and autism genes from the most up-to-date WES study, to the best of our knowledge, for either disorder in order to validate our results on a relatively unbiased gene set (Fig. 1A). In both the gene and phenotype enrichment analysis, we found a similar trend as the previous analysis on the multiplex network with all epilepsy- and autism-associated genes (which we will refer to as the larger multiplex network). That is, there is one module most specific to epilepsy and one module most specific to autism, both being the only modules significantly enriched in common genes (FDR = 3.90e−06 and FDR = 0.0186, respectively) (Fig. 6A). Moreover, the epilepsy-focused module, module 2, consisting of 30 genes, is also enriched in HC common genes (FDR = 2.64e−08) as well as WES common genes (FDR = 0.0113) that are both in the epilepsy and autism WES gene lists, relative to other modules. In the WES multiplex network, module 2 was the only module enriched in Epilepsy 1 genes (FDR = 5.74e−14) and it was the most strongly enriched in epilepsy phenotypes relative to other modules (Fig. 6B). Module 2 was also enriched in Autism 1 genes (FDR = 0.0119), WES autism genes (FDR = 0.0149), schizophrenia genes (FDR = 1.17e−04), BD genes (FDR = 7.55e−04), and BEGs (FDR = 3.01e−08). The most significantly enriched GO biological process for the module is ion transmembrane transport (GO: 0034220, FDR = 1.20e−17, 22/30 genes in the module). Twenty-six out of the thirty genes in module 2 also exist in the larger multiplex network; 20/26 (77%) are in module 3, 4/26 in module 6 (15%), and 1 in each of modules 1 and 13, in the larger network. Thus, module 2 in the WES multiplex network corresponds to module 3 of the larger multiplex network, sharing the same GO biological process enrichment, genes, and phenotypes (Fig. 7A,C). Table S3 in supplementary Excel file contains additional information on the GO and HPO enrichments of each WES module as well as the subgroups of epilepsy and autism genes in the module.

Along with module 2 in the WES multiplex network, module 7, consisting of 12 genes, was also enriched in common genes (FDR = 0.0186). It was also enriched in Autism 1 (FDR = 0.0186) and Epilepsy 3 (FDR = 4.62e−03) genes and it was the most strongly enriched in autism phenotypes (Fig. 6B) relative to other modules. The most significantly enriched GO biological process for the module was modulation of synaptic transmission (GO: 0050804, FDR = 0.0816, 4/12 genes in the module). Eight out of the twelve genes in module 7 also exist in the larger multiplex network: 3/8 (38%) in each of modules 6 and 4, and 1 in each of modules 1 and 8. Module 7 in the WES multiplex network corresponds most closely to module 6 of the larger multiplex network because of sharing similar GO biological process enrichment (related to synaptic signaling) and autism gene and phenotype enrichment (Fig. 7B,D). Even with the limited number of genes in the WES multiplex network (294 genes) and small module sizes, we obtain a similar enrichment pattern, namely we show that module 2 and module 7 in the WES multiplex network correspond to modules 3 and 6, respectively, of the larger multiplex network. The results support that modules 3 and 6 of the larger multiplex network, while primarily associated with epilepsy and autism respectively, contain a significant number of common genes and their representative biological processes (ion transmembrane transport and synaptic signaling, respectively) are relevant to epilepsy and autism etiology.

### Prioritizing candidate epilepsy and autism genes

We can also prioritize candidate genes using the module characterizations. Module 3 of the multiplex network and module 2 of the WES network, showed significant enrichment in Epilepsy 1 genes (genes directly causing epilepsy) and epilepsy phenotypes compared to other modules in the network. Looking at the overlap of these two modules which consists of 20 genes, *ANK2*, *CACNA1E*, *CACNA2D3*, *GRIA2,* are the only autism-specific genes, meaning that they were not identified as epilepsy genes in Wang et al. (2017) (Fig. 1C)^10^. It can be hypothesized that these genes are also associated with epilepsy because they fall in a high confidence epilepsy module that is also enriched in common genes (genes associated with both epilepsy and autism). The only other autism-specific gene in module 2 of the WES network is *DLG4*, which belongs to module 6 of the larger multiplex network. Module 6 is also enriched in common genes as well has some epilepsy phenotypes relative to other modules, so it can also be hypothesized that *DLG4*, although only labelled as an Autism 1 gene, is also associated with epilepsy.

To validate the novel predictions, we performed literature review to identify evidence supporting the potential role of these genes in epilepsy. *ANK2*, which encodes for the ankyrin-B protein, a member of the ankyrins family, was predicted as a novel epilepsy-related gene by a recent network-based study using a random walk with restart algorithm^32^. A recent study showed that an *ANK2* variant is associated with seizure possibly through its interactions with the voltage-gated Ca_V_2.1 calcium channel^33^. De novo pathogenic variants of *CACNA1E*, which encodes the α_1_-subunit of the voltage-gated Ca_V_2.3 channel, were recently identified to cause developmental and epileptic encephalopathy^34,35^. De-novo variants of *GRIA2*, which encodes the GluA2 subunit of AMPA type ionotropic glutamate receptors, have been to be shown to cause neurodevelopmental disorders^36^. A recent study demonstrated that an engineered mutation in *GRIA2* caused seizure vulnerability as well as learning and memory impairments^37^. *DLG4*, which encodes a scaffold protein in the postsynaptic region, was predicted as a candidate epilepsy gene in a random walk-based module prediction study because it showed up frequently and exclusively in modules with epilepsy genes^22^. Furthermore, *DLG4* was reported as a candidate gene in the epilepsy WES study^38^. There is no literature specifically supporting the role of *CACNA2D3* in causing seizures or epilepsy, but it encodes for a member of the α_2_δ subunit family of voltage-gated calcium channels, which have a role in epilepsy and antiepileptic drug pharmacology^39,40^. Therefore, there are literature supporting the role of the five proposed candidate genes in epilepsy.

Moreover, common genes collectively have a significantly higher centrality in the network than epilepsy- or autism- specific genes (Fig. S4). Therefore, within our two prioritized modules, modules 3 and 6, the genes can be further prioritized by their degree in the PPI and phenotype network and well as their betweenness centrality (Tables 2 and 3). Because module 3 is an epilepsy-focused module, autism-specific genes in the module may also be associated with epilepsy. Similarly, while module 6 is an autism-focused module, epilepsy-specific genes in the module may also be associated with autism. The genes in these two modules should be further examined to understand the shared genetic etiology of epilepsy and autism.

**Table 2:** Highest centrality genes in module 3.

Top 20 genes by average degree	PPI degree	Phenotype degree	Annotation	Top 20 genes by average BC	PPI BC	Phenotype BC	Annotation
CALM2	32	139	Epilepsy 3	CALM2	0.00665306	0.01452167	Epilepsy 3
CACNG2	33	70	Epilepsy 3	**AKAP9**	0.00902041	0.00222203	Autism 2
**AKAP9**	47	53	Autism 2	**NRCAM**	0.00154433	0.00815726	Autism 3
CACNA2D2	16	73	Epilepsy 4	KCNAB2	0.00673046	0.0024144	Epilepsy 4
**CACNA1C**	35	49	Autism 1	CACNA2D2	0.00056149	0.00762001	Epilepsy 4
**KCNS3**	3	68	Autism 2	CACNG2	0.00262347	0.00493458	Epilepsy 3
**CACNA2D3**	10	61	Autism 2	**CACNA1C**	0.00619087	0.00080327	Autism 1
KCNB1	5	66	Epilepsy 1, Autism 1	CACNA1A	0.00647452	0.00040586	Epilepsy 1, Autism S
KCNA2	13	58	Epilepsy 1	CNTN2	0.00026388	0.00651281	Epilepsy 1
KCND2	7	64	Epilepsy 4, Autism 3	GABRB1	4.39E−05	0.00625521	Epilepsy 1
**GRIA2**	32	38	Autism 2	**CACNA2D3**	7.99E−05	0.00614434	Autism 2
KCNAB1	15	55	Epilepsy 4	**USH2A**	0.00175342	0.00408082	Autism 3
SCN3A	9	60	Epilepsy 4	ANK3	0.00210367	0.00350903	Epilepsy 3, Autism 1
GABRA6	13	54	Epilepsy 4	FGF12	6.39E−05	0.0051396	Epilepsy 1
**CACNA1B**	20	47	Autism 3	GPHN	0.0046279	0.00043612	Epilepsy 3, Autism 2
**KCNK7**	0	66	Autism 3	**HTR3A**	0.00144429	0.00358634	Autism 3
KCNMB3	3	62	Epilepsy 4	KCNB1	0.00017102	0.00484786	Epilepsy 1, Autism 1
**GABRA4**	13	52	Autism 3	CHRNA4	0.0037866	0.00120251	Epilepsy 1
CACNA1D	22	41	Epilepsy 3, Autism 2	**ACHE**	0.00277761	0.00141233	Autism 2
**GABRG3**	10	51	Autism 2	**CACNA1E**	0.0008944	0.00322882	Autism 2

The top 20 genes in module 3 are listed and ranked by average degree and average betweenness centrality (BC). Genes that are autism-specific are bolded since module 3 is an epilepsy-focused module.

**Table 3:** Highest centrality genes in module 6.

Top 20 genes by average degree	PPI degree	Phenotype degree	Annotation	Top 20 genes by average BC	PPI BC	Phenotype BC	Annotation
DLG4	75	87	Autism 1	DLG4	0.02611288	0.00199529	Autism 1
GRM5	47	49	Autism 3	SNAP25	0.01245612	0.00012814	Epilepsy 4, Autism 3
GRIA1	45	40	Autism 2	GRIA1	0.00943414	0.00236783	Autism 2
**DLG3**	37	19	Epilepsy 3	GRM5	0.00310818	0.00609329	Autism 3
SNAP25	46	7	Epilepsy 4, Autism 3	SHANK1	0.00068914	0.0084605	Epilepsy 4, Autism 2
NRXN2	25	27	Autism 1	**SNRPN**	0.001995	0.00575377	Epilepsy 3
**GRM1**	49	2	Epilepsy 3	CHD8	0.00628398	0.00034323	Epilepsy 3, Autism 1
NRXN3	24	26	Autism 1	FMR1	0.00591127	0	Epilepsy 3, Autism 1
SHANK1	25	25	Epilepsy 4, Autism 2	DLG1	0.00499386	0.00065879	Autism 3
STXBP1	24	25	Epilepsy 1, Autism 1	GRIK5	0.00022519	0.0047488	Autism 2
NLGN4X	21	27	Autism 2	**GRM1**	0.0049405	0	Epilepsy 3
DLGAP1	21	27	Autism 2	CNKSR2	3.16E−05	0.00450633	Autism 2, Autism S
NLGN2	22	25	Autism 1	HCFC1	0.00444098	1.86E−05	Epilepsy 3, Autism S
SHANK2	20	27	Autism 1	USP9X	0.0038062	0.00032409	Epilepsy 3, Autism S
NLGN1	22	24	Epilepsy 4, Autism 2	STX1A	0.00388987	0	Autism 3
CNKSR2	11	35	Autism 2, Autism S	STXBP1	0.00283952	0.00090111	Epilepsy 1, Autism 1
NLGN3	21	23	Autism 1	SYT1	0.00352268	0	Autism S
DLGAP2	19	25	Autism 3	**GDI1**	0.00331726	1.86E−05	Epilepsy 3
**SNRPN**	17	25	Epilepsy 3	CDKL5	0.00139387	0.00166681	Epilepsy 1, Autism 1
STX1A	34	8	Autism 3	NLGN2	0.00148443	0.00156034	Autism 1

The top 20 genes in module 6 are listed and ranked by average degree and average betweenness centrality (BC). Genes that are epilepsy-specific are bolded since module 6 is an autism-focused module.

## Discussion

In our study, we used a multiplex network of epilepsy- and autism-associated genes to elucidate the relationship between the two disorders, epilepsy and autism, that often co-occur. The multiplex network contains a gene-PPI layer and a gene-phenotype layer. PPI networks have recently become widely used to understand the molecular basis behind human diseases^28^. PPI networks can be used to discover new disease genes, study characteristics of the disease gene network, identify disease-related and functional sub-networks, and help classify diseases using network properties^41^. While it is often assumed that PPI modules overlap with disease phenotypes modules, we demonstrated this observation by comparing the gene-PPI layer to the gene-phenotype layer of the multilayer network of expert-compiled and well-established epilepsy- and autism-associated genes (Fig. 2). While there is a significant overlap of PPI modules and phenotype modules, combining both layers in a multiplex network formally factors in both functional similarities, through PPI interactions, and similarities in phenotypic manifestations of genes, which we wanted to capture in the multiplex modules. Furthermore, important genes such as *SHANK3* and *NLGNY* that could not be found in the STRING PPI database, were sorted into appropriate modules using their relationships in the gene-phenotype layer of the multiplex network.

Because the global information of the entire multiplex network of 1707 genes is too general, we used a multiplex community detection to cluster highly interacting and similar genes together in modules. We showed that genes within a module interact with each other in the same biological processes and have specific gene and phenotype enrichments (Figs. 4, 5, Tables S2, S3). In particular, we found that modules 3 and 6 are significant in the epilepsy-autism multiplex network because of their enrichment in common genes, BEGs, high confidence epilepsy and autism genes, as well as epilepsy and autism phenotypes relative to other modules. Similar modules showed the same patterns of enrichment in a multiplex network constructed using epilepsy and autism genes solely from WES studies.

Module 3 in the epilepsy-autism multiplex network represents genes involved in ion transmembrane transport; many of the genes in this module encode for subunits of ion channels. Several monogenetic epilepsies are associated with mutations in genes encoding for ion channels^10,42–44^, and ion channel dysfunctions are also linked to susceptibility to autism, as well as bipolar disorder, schizophrenia and other neuropsychiatric disorders^45^, which explains their enrichment in module 3 and the comparable module 2 of the WES multiplex network. This module also contains genes encoding GABA receptors and nicotinic acetylcholine receptors, which are known to be related to epilepsy, autism, and other brain disorders^46,47^. Module 6 contains genes involved in synaptic signaling, a shared pathway between epilepsy and autism that has been supported by several studies^20,22^. This module contains genes encoding ionotropic and metabotropic glutamate receptors, and families of genes involved in the regulation and maintenance of synapses, such as *DLGAP*, *NRXN,* and *NLGN*, which are all known to be related to brain disorders^48–51^. Furthermore, module 6 was enriched in genes involved in intellectual disability, the severity of which is related to epilepsy risk in autism^52^. From modules 3 and 6 we prioritize *ANK2*, *CACNA1E*, *CACNA2D3*, *GRIA2*, and *DLG4*, genes not included the list of epilepsy genes in Wang et al. (2017)^10^, as candidate epilepsy genes because of their overlap with the epilepsy-focused module 2 of the WES network. Moreover, we find that common genes have greater centrality in both the PPI and phenotype network, so we list the highest centrality genes in modules 3 and 6 in Tables 2 and 3, respectively. We recommend these genes for further review as potential common genes.

We also wish to discuss several limitations of the current study. First, our analysis, including the epilepsy and autism subgroups, depends on previously compiled list of disease genes from human experts, and therefore it is biased towards well studied genes and probably biased towards genes with more known interaction partners in the PPI or phenotype network. In other words, genes with high degree may be studied more because they are related to disease, creating bias in the number of connections they have^53^. This is something that should be considered when interpreting results, since less studied genes could still be important contributors to both diseases. It also highlights the utility of the module-based analysis that groups genes with similar features together so lesser-known genes can be characterized. The bias limitations can be reduced as the datasets are further validated and improved in the future.

Another limitation comes from our generalization of genetic etiologies to discrete genes in the multiplex network. This generalization does not account for how different mutations within a gene can result in different phenotypes^54^. For example variations in *SCN1A*, one of the most commonly studied epilepsy genes, can result in a range of epilepsy syndromes^55^, from generalized epilepsy with febrile seizures plus (GEFS +) to Dravet syndrome (DS), which is associated with intellectual disability and autism^56^. Moreover, due to incomplete penetrance and variable expressivity^54^, similar mutations may result in varying phenotypes when compared across individuals^57^. Moreover, unlike epilepsy which manifest as characteristic seizures, the phenotypic spectrum of autism makes its diagnosis especially challenging^58^, which is important to keep in mind when studying the disorder. In the future, we should incorporate additional genotype–phenotype analyses at the individual patient level. These analyses will help validate our current model of modules and pathways involved in both epilepsy and autism.

Beyond attempting to overcome these limitations, there are also other ways to expand on this research. It has been shown that many neurological disorders have a common genetic etiology^54^. Therefore, beyond epilepsy and autism, other neurological disorders like depression, anxiety, obsessive compulsive disorder (OCD), and attention deficit hyperactivity disorder (ADHD) should also be explored in relation to epilepsy and autism, when we have a reasonable number (> 100) high confidence genes for these disorders. Finally, there is a known deep genetic relationships between neurodevelopmental disorders and cancer^59^ and given that the Cancer Gene Census already documents several hundred cancer-relevant genes, it would be of interest to perform similar analysis on neurodevelopmental disorders and cancer (a preliminary analysis shows that 105/723 of the genes in Cancer Gene Census are in the SFARI gene list, confirming the striking genetic connection between these two distinct conditions). In summary, while we acknowledge the exploratory nature of this current study, the approaches presented in the manuscript enables these future research directions and may generate novel insights into the shared genetic etiology between multiple well-studied diseases.

## Methods

### Data resource and description

#### Epilepsy- and autism-associated genes

We collected 977 genes, including gene clusters, associated with epilepsy from the expert-compiled list from Wang et al. (2017)^10^. These genes were manually curated and examined from multiple genetic databases and represent genes are directly related to epilepsy, or indirectly lead to epilepsy through influence on the central nervous system or other systems; the subgroups of epilepsy genes are defined in Table 1. These 977 epilepsy-associated genes were mapped to 999 genes in the multiplex network. The number of genes increased because gene clusters were separated into individual genes. We also collected 913 autism-associated genes from the Simons Foundation Autism Research Initiative (SFARI) Gene (access date: January 5, 2020), a community-driven knowledgebase of autism spectrum disorder^60^. SFARI Gene has the evidence and the strength of the genetic association for each gene; subgroups of autism genes are defined in Table 1. These 913 autism-associated genes were also mapped to the multiplex network. In total there were 1707 epilepsy- and autism-associated genes.

#### Whole exome sequence data from other independent studies

In additional analyses, we also used the most updated whole-exome sequencing (WES) data we could find for each of autism and epilepsy in order to test whether our results were also applicable to a less biased gene set^38,61^. The 102 autism genes with FDR <  = 0.1 from the autism WES study (see Table S2 in Satterstrom et al., 2020) were used as the autism gene set. The top 200 most significant epilepsy genes outputted by the gene burden test from the epilepsy WES study (see Table S17 in Feng et al., 2019) were used as the epilepsy gene set.

#### Genes associated with other brain disorders

The gene lists for schizophrenia, bipolar disorder, and intellectual disability were retrieved from a paper by Wang et al. (2018)^62^. The schizophrenia gene list originally comes from the SZgene^63^ database and a GWAS study^64^. The bipolar disorder gene list originally comes from the BDgene database^65^. The intellectual disability gene list originally comes from BrainSpan and are documented in a previous publication by the same authors^66^.

### Network generation

#### Protein–protein interaction (PPI) network

We mapped all 999 epilepsy and 913 autism-associated genes to the STRING PPI database (version 11)^67^ for *Homo sapiens* to generate the epilepsy-autism PPI network. The interactions in STRING consist of known and predicted interactions including direct physical and indirect functional associations evaluated from experiments, predictions, and knowledgebase. A node with degree zero was created for genes that did not exist in STRING, since they may have non-zero degree in the phenotype network. A total of 1707 epilepsy- and autism-associated genes were represented as nodes in the PPI network. Interactions from the STRING database were used as edges between the nodes, at first weighted by their confidence score determined by STRING. The edges in the PPI network were thresholded at a weight of 700, representing high confidence connections^67^, so that only edges with a weight of at least 700 were used in the PPI network, and the resulting PPI network had binary edge weights.

#### Phenotype network

The phenotype network was generated based on a phenotypic similarity score between pairs of genes representing how similar they are in their phenotype associations. For each of the 1707 epilepsy- and autism-associated genes, all phenotype associations were retrieved from the Phen2Gene knowledgebase, a comprehensive and standardized database of phenotype-gene associations that standardizes phenotypes using the Human Phenotype Ontology (HPO)^30,31^. Only HPO IDs under the parent “Phenotypic abnormality” (HP:0000118) were used in order to retrieve the most relevant phenotypic information. The Phen2Gene knowledgebase contains a score for each HPO-gene relationship, representing the strength of their association, and the top 1000 and top 500 scoring genes for each HPO ID were considered, respectively, to generate the phenotype network in the larger multiplex and WES multiplex network (Table S1). For each gene, a corresponding phenotype vector was created where the length was the total number of HPO IDs in the Phen2Gene database. Each vector element represents an HPO ID and the value is the phenotype-gene association score weighted by the skewness of the HPO ID (described in the original Phen2Gene paper). The phenotypic similarity score between two genes is then the cosine similarity between their corresponding pair of phenotype vectors. An edge between the two genes was added to the phenotype network if their phenotypic similarity score was above a certain threshold. The threshold was determined by randomly shuffling the two phenotype vectors 1000 times, sorting the phenotypic similarity scores, and choosing the 10^th^ largest value (representing a p-value of 0.01). A significance level of 0.01 was chosen in order to uncover the modular nature of the phenotype network by keeping the most important edges (Table S1). The resulting phenotype network had binary edge weights.

#### Multiplex network and clustering algorithm

The multiplex network represents a multilayer network where the same nodes exist in each layer; the network encodes both the PPI relationships and phenotype relationships between the genes in the network. The gene-PPI-phenotype multiplex network was created by stacking the PPI network and gene-phenotype network layers, generated as detailed in the previous sections, such that each node in one layer is connected to itself in the other layer.

The Louvain algorithm is a modularity maximization approach that is commonly used to detect modules in a network and has been shown to perform well on biological networks^68^. For the individual PPI and phenotype layers, the Louvain algorithm was used to maximize the modularity, H, defined by:

$$H= \frac{1}{2m}{\sum }_{c}({e}_{c}-\gamma \frac{{K}_{c}^{2}}{2m})$$

Here, ${e}_{c}$ is the total number of edges in community $c$, $m$ is the total number of edges in the network, and ${K}_{c}$ is the sum of the degree of the nodes in community $c$. To maximize modularity, which the Louvain algorithm is useful for, then is to maximize the difference between the actual number of edges and the expected number of edges in a community. In the equation, $\gamma$, is the resolution parameter which controls the size of the communities. The Louvain algorithm was applied 1000 times with different random seeds at a range of resolutions and the partition with the globally optimal modularity was chosen (Fig. S3). The Louvain algorithm can be easily extended to be applicable to a multiplex network. In this case the overall modularity, which the algorithm will try to maximize, is the sum of the modularity of each layer weighted by some constant:

$$H={{w}_{ppi}H}_{ppi}+{{w}_{phenotype}H}_{phenotype}$$

We set both layers to have equal weights in order to have equal contribution from the PPI and phenotype layers. The louvain-igraph Python package was used to run the Louvain algorithm (https://louvain-igraph.readthedocs.io/).

### Gene Ontology enrichment analysis

Gene Ontology (GO) is a tool that helps unify understanding of biological functions of genes and proteins in eukaryotes^69^. After modularity detection, the genes within each prescribed module were collectively analyzed using GO for their biological processes, defined as a biological objective to which the genes in the module contribute. The Database for Annotation, Visualization and Integrated Discovery (DAVID) was used to retrieved GO terms and their enrichment using a Fisher’s exact test^70^. DAVID also reports the FDR for each enrichment.

### Differentially expressed gene analysis in brain tissues

To further evaluate whether epilepsy- and autism-associated genes are correlated with gene expression within human brain tissue, we downloaded RNA-Seq data of Genotype-Tissue Expression (GTEx) from UCSC Xena^71^. As there are 1141 samples from post-mortem, multi-region brain tissue, we randomly selected a comparable size of control samples (n = 1011) from whole blood, muscle and nerve tissue. We performed differential expression genes analysis to compare brain and control tissues using the Limma-voom method in the edgeR package^72^. The pattern of identified brain-enriched genes (BEGs) are shown in Fig. S5.

### Statistical analyses and network properties

To measure the enrichment of the different subgroups of epilepsy-associated genes, autism-associated genes, and common genes (associated with both disorders) in each module, gene enrichment analyses^73^ were performed for each gene group, assuming a hypergeometric distribution. This test measures the enrichment of a given group of genes in a module compared to what would be expected by a random distribution of genes among the modules, which is the null hypothesis. The p-values were calculated using the hypergeom function from scipy.stats version 1.4.1. False discovery rate correction was applied using the multitest.fdrcorrection function from statsmodel.stats version 0.12.0 (https://www.statsmodels.org/stable/index.html). To measure the enrichment of different HPO IDs in each module, an empirical p-value was determined by computing the mean gene-phenotype association score for each HPO ID over the genes in the module and comparing it to the mean of 10,000 trials using *n* genes in the Phen2Gene knowledge base, where *n* is the size of the background. The phenotype enrichment was also calculated using annotated gene-HPO relationships from hpo.jax.org. The hypergeometric test was used to determine the p-value and corrected using false discovery rate, similar to the gene enrichment tests.

Several network statistics were used in this paper. The Community Discovery Library (CDlib)^74^ version 0.1.9 was used for modularity scoring. The Newman-Girvan modularity score represents the difference of intra-module edges with the expected number of such edges according to a null model^75^. The python-igraph package version 0.8.3 (https://igraph.org/python/) was used to construct the multiplex network. NetworkX version 2.5 (https://networkx.org/) was used to construct the individual network layers, calculate the degree of the nodes (the number of adjacent nodes connected by an edge), generate random networks with a given degree distribution, and calculated the shortest path betweenness centrality of a node. The betweenness centrality of a node measures the number of shortest paths within the network that go through that node^76^. The formula for the betweenness centrality of a node, $v$, is as follows^76^:

$$B\left(v\right)={\sum }_{s,t \in V}\frac{\sigma (s,t|v)}{\sigma (s,t)}$$

where $V$ is the set of nodes in the network, $\sigma (s,t)$ is the number of shortest paths between $s$ and $t$, and $\sigma (s,t|v)$ is the number of those shortest paths passing through node $v$. If $s=t$, $\sigma \left(s,t\right)=1$ and if $v\in \{s,t\}$, $\sigma \left(s,t|v\right)=0$.

## Conclusion

It is well known that autism spectrum disorders (ASDs) and epilepsy commonly co-occur, but the underlying genetic connection between the two disorders requires further research, which once better understood, will facilitate the implementation of precision medicine for both diseases. In this study, we detect modules in a multilayer network of epilepsy- and autism-associated genes representative of subgroups of epilepsy genes related through involvement in similar biological processes and having similar genotypic and phenotypic features. The protein–protein interaction (PPI) layer of the multiplex network is complementary to the gene-phenotype layer and when integrated in a multiplex network allows the identification of genetic modules that are highly connected through PPI interactions and share similar phenotypic associations. We were able to identify two modules enriched in common genes, genes associated with both epilepsy and autism, representing shared biological processes disrupted in the disorders. The first module, representing ion transmembrane transport, is more epilepsy-focused in terms of genotypic and phenotypic enrichments while the second module is more autism-focused and represents synaptic signaling and synapse regulation and maintenance. Two similar modules were identified in a multiplex network constructed using epilepsy and autism genes from WES studies. We prioritize the following candidate epilepsy genes, which are found in the epilepsy-focused modules of both the larger multiplex network and WES network: *ANK2*, *CACNA1E*, *CACNA2D3*, *GRIA2*. Another candidate epilepsy gene is *DLG4*, which is in epilepsy focused module of the WES network and autism-focused module of the larger phenotype network. These genes, although associated with ASD in the SFARI database, were not listed as epilepsy candidates in Wang et al. (2017)^10^. The two modules are also enriched in genes upregulated in brain tissue, bipolar disorder-associated genes, and intellectual disability genes, so they could explain comorbidities of epilepsy and autism with other neuropsychiatric and neurodevelopmental disorders. The two modules warrant further investigation in the future, as well as the less epilepsy- and autism-specific modules that may have more indirect relationships with the disorders. The computational and analytical approaches used in our study may be also applied in similar future studies to study the genetic connection between different human diseases.

## Supplementary information

Supplementary Information 1.

Supplementary Information 2.

Supplementary Information 3.

## Data Availability

All the code and data sets are organized in a computational workflow available at https://github.com/WGLab/epilepsy-autism-multiplex-network for reproducible research.
