# Supplementary material for: Multiplex gene and phenotype network to characterize shared genetic pathways of epilepsy and autism
Source: Sci Rep. 2021 Jan 13;11:952. doi: 10.1038/s41598-020-78654-y (PMC7806931; doi:10.1038/s41598-020-78654-y)
Supplement: Supplementary file 1 — Supplementary Information 1. [file 41598_2020_78654_MOESM1_ESM.pdf]

Supplemental Figures

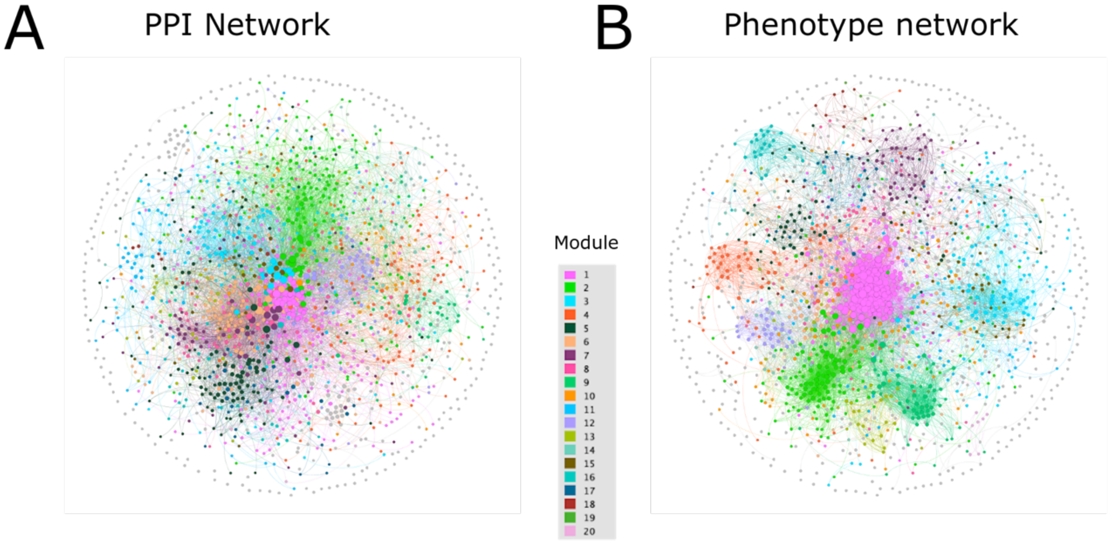

Figure S1. Modules in the PPI and phenotype network layers of the epilepsy-autism multiplex network. Nodes belonging to the top 20 largest modules are colored based on the module they belong to. Only PPI modules and edges in the PPI network layer are shown in **(A),** and only phenotype modules and edges in the phenotype network are shown in **(B)**. The position of each gene is identical in both figures and in **Figure 1A**. The figure was generated using Gephi version 0.9.2, a graph visualization software.

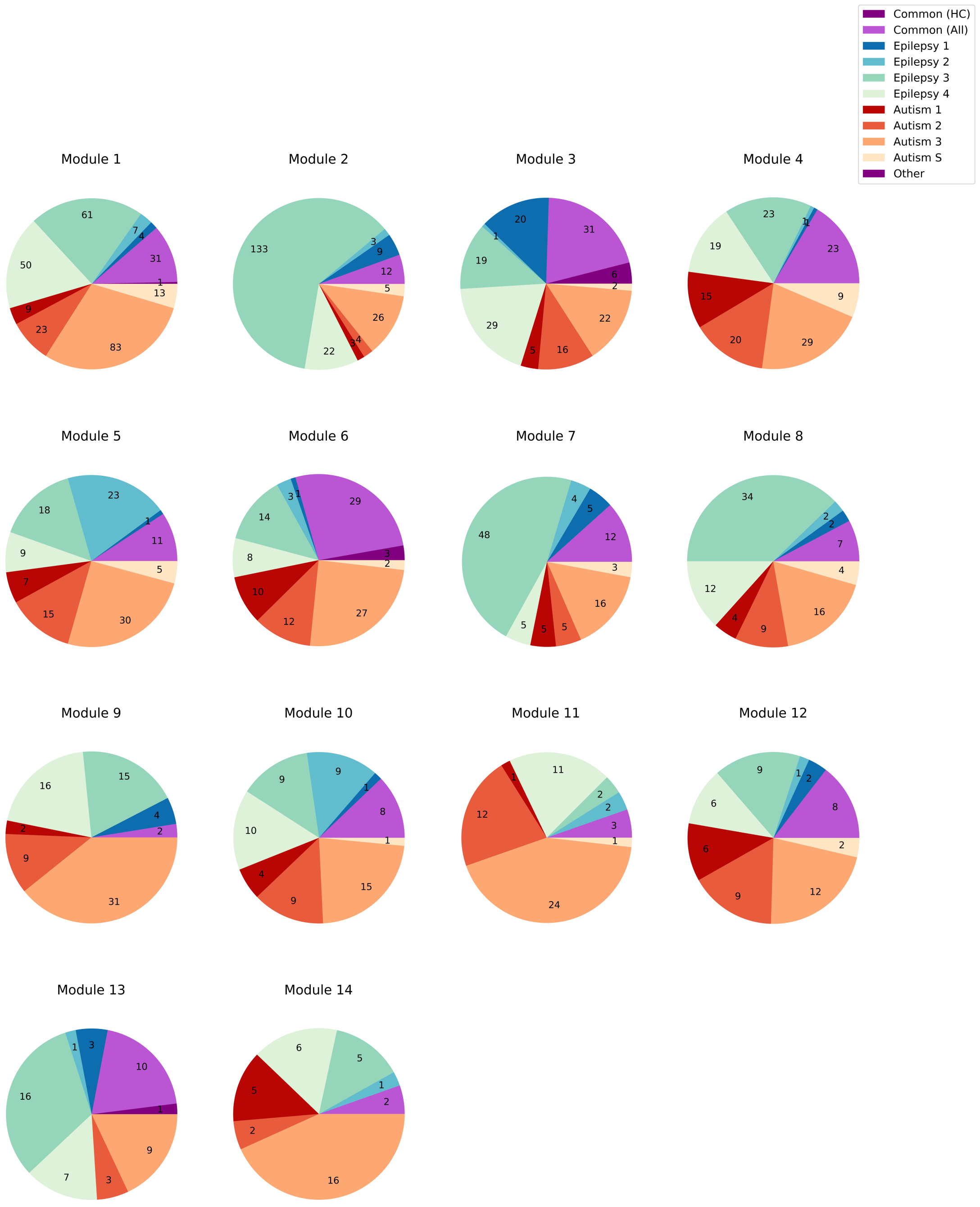

Figure S2. Composition of the 14 largest modules in the epilepsy-autism multiplex network. For the 14 largest modules in the multiplex network, counts are shown for subgroups of epilepsy and autism genes as well as common genes. Common (HC)=genes that are both in the epilepsy 1 subgroup and autism 1 subgroup (high confidence), Common (All)=all genes in an epilepsy subgroup and autism subgroup. If a gene falls under multiple classifications, it is only classified under one of them in the priority shown from top to bottom in the legend.

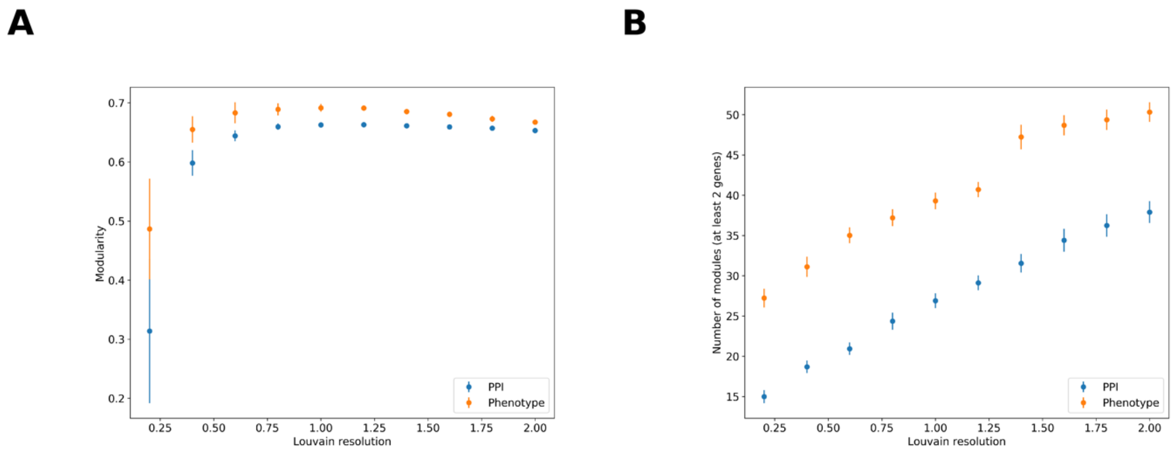

Figure S3. Louvain single layer community detection resolution. To determine what resolution of the Louvain algorithm to use to generate the modules in the PPI and phenotype networks, several resolutions were tested. The Louvain algorithm was run 1000 times at each resolution. The plots show **(A)** average (Newman-Girvan) modularity ± the standard deviation and **(B)** number of communities (with at least 2 genes) ± the standard deviation as a function of the resolution. A resolution of 1, which maximizes the modularity, was chosen.

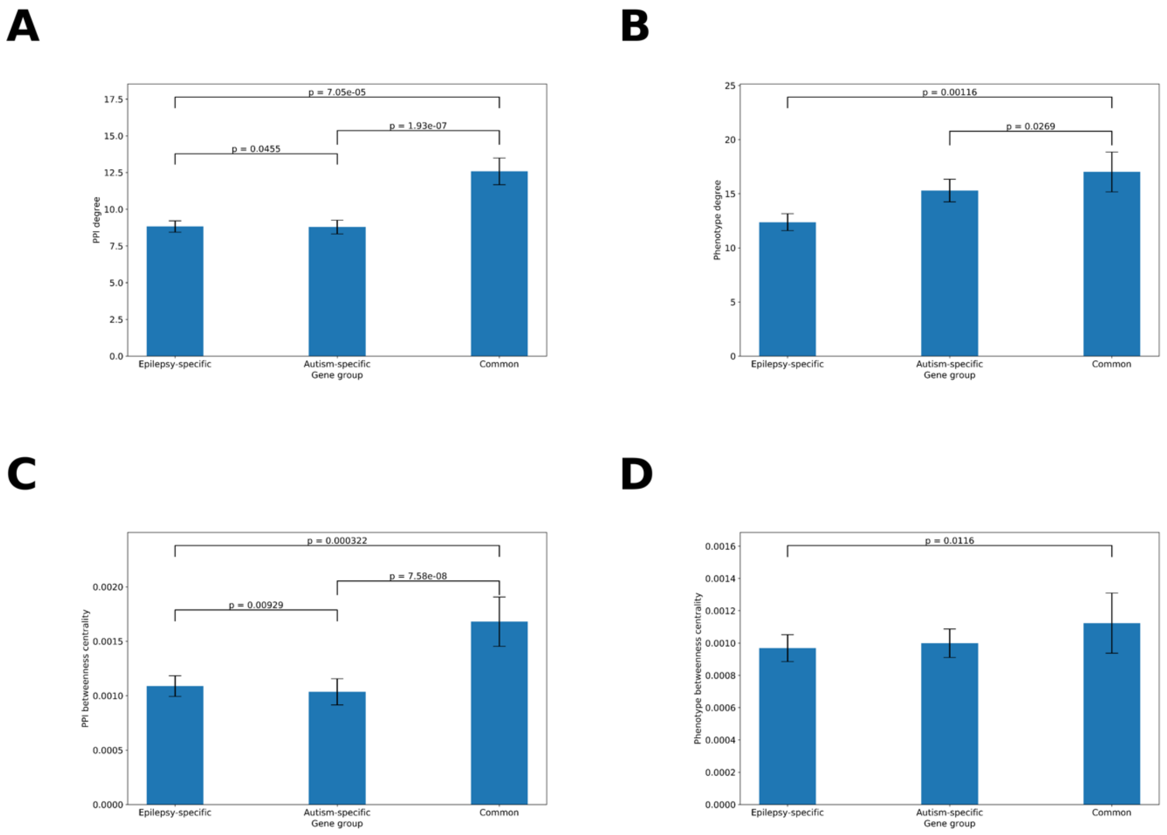

Figure S4. Centrality comparison between epilepsy-specific, autism-specific, and common genes. For each network statistic--PPI (network) degree, phenotype degree, PPI betweenness centrality, phenotype betweenness centrality--the Kruskal-Wallis H test (one-way non-parametric ANOVA) was performed, where the type of gene (epilepsy-specific, autism-specific, or common) was the dependent variable and the network statistic was the dependent variable, to determine whether the type of gene had a significant effect on the network statistic. After confirming the effect was significant, a Mann-Whitney U test with Bonferroni correction was performed as a post hoc method to compare the statistical significance between groups and the p-value is displayed in the figure. The bar plots represent mean ± standard error of the mean. ‘Common genes’ represents genes that are both epilepsy- and autism-associated, ‘epilepsy-specific’ is the difference of epilepsy-associated genes and common genes, and ‘autism-specific’ is similarly the difference of autism-associated genes and common genes. The statistical tests were performed with scipy.stats version 1.4.1 (https://docs.scipy.org/doc/scipy/reference/stats.html) and scikit-posthocs version 0.6.1 (https://scikit-posthocs.readthedocs.io/en/latest/).

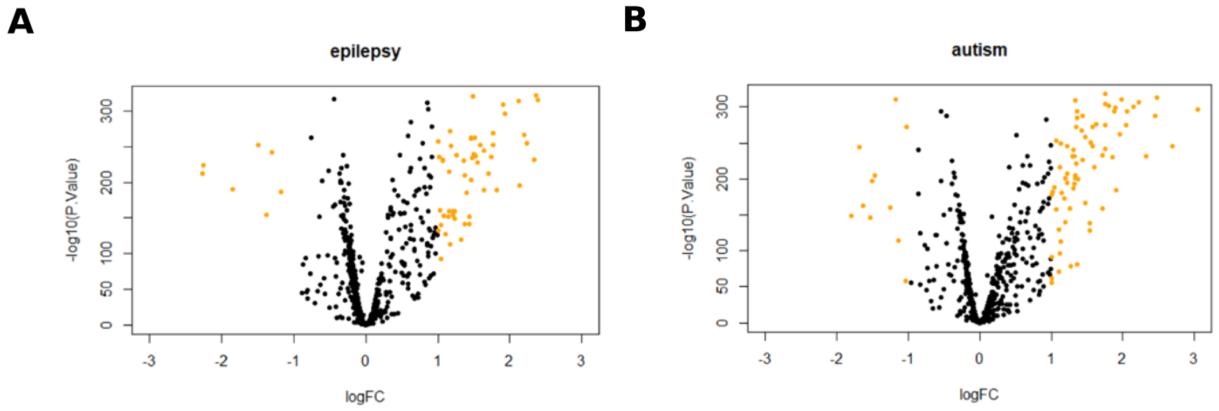

Figure S5. Differential expression of epilepsy and autism genes in brain tissue. The analysis was performed using the R package edgeR and plots were created by the R package ggplot2 (https://ggplot2.tidyverse.org).
