# Supplementary material for: Multiplex gene and phenotype network to characterize shared genetic pathways of epilepsy and autism
Source: Sci Rep. 2021 Jan 13;11:952. doi: 10.1038/s41598-020-78654-y (PMC7806931; doi:10.1038/s41598-020-78654-y)
Supplement: Supplementary file 2 — Supplementary Information 2. [file 41598_2020_78654_MOESM2_ESM.docx]

Integrative network analysis to characterize shared genetic pathways of epilepsy and autism

Jacqueline Peng ^1^, Yunyun Zhou ^2^, Kai Wang ^2,3^

^1^ Department of Bioengineering, University of Pennsylvania, Philadelphia, PA 19104, USA

^2^ Raymond G. Perelman Center for Cellular and Molecular Therapeutics, Children's Hospital of Philadelphia, Philadelphia, PA 19104, USA

^3^ Department of Pathology and Laboratory Medicine, University of Pennsylvania, Philadelphia, PA 19104, USA

Keywords: epilepsy, autism, autism spectrum disorder, co-morbidity, Human Phenotype Ontology, network analysis

Correspondence should be addressed to KW: [wangk@email.chop.edu](mailto:wangk@email.chop.edu)

**Legend for supplementary tables**

**Table S1**: Modularity analysis for phenotype network generation. To keep the most important edges, we choose 0.01 as the significant cutoff to uncover the modular nature of the phenotype networks for all genes and WES genes.

**Table S2**: Gene Ontology and Phenotype enrichment analysis for each module and subgroups of epilepsy and autism genes in the multiplex module for all genes.

Table S3: Gene Ontology and Phenotype enrichment analysis for each module and subgroups of epilepsy and autism genes in the multiplex module for WES genes.
